# Stroke population–specific neuroanatomical CT-MRI brain atlas

**DOI:** 10.1007/s00234-021-02875-9

**Published:** 2022-01-30

**Authors:** Tina Kaffenberger, Vijay Venkatraman, Chris Steward, Vincent N. Thijs, Julie Bernhardt, Patricia M. Desmond, Bruce C. V. Campbell, Nawaf Yassi

**Affiliations:** 1 The Florey Institute of Neuroscience and Mental Health, University of Melbourne, 245 Burgundy Street, Heidelberg, VIC 3084 Australia; 2 Department of Radiology, Royal Melbourne Hospital, University of Melbourne, Parkville, VIC Australia; 3 Department of Neurology, Austin Health, Heidelberg, VIC Australia; 4 Department of Medicine and Neurology, Royal Melbourne Hospital, Parkville, VIC Australia; 5 Melbourne Brain Centre, University of Melbourne, Parkville, Melbourne, VIC Australia; 6 Population Health and Immunity Division, The Walter and Eliza Hall Institute of Medical Research, Parkville, VIC Australia

**Keywords:** Computed tomography (CT), Magnetic resonance imaging (MRI), Stroke, Population specific, Template, Neuroanatomical atlas

## Abstract

**Purpose:**

Development of a freely available stroke population–specific anatomical CT/MRI atlas with a reliable normalisation pipeline for clinical CT.

**Methods:**

By reviewing CT scans in suspected stroke patients and filtering the AIBL MRI database, respectively, we collected 50 normal-for-age CT and MRI scans to build a standard-resolution CT template and a high-resolution MRI template. The latter was manually segmented into anatomical brain regions. We then developed and validated a MRI to CT registration pipeline to align the MRI atlas onto the CT template. Finally, we developed a CT-to-CT-normalisation pipeline and tested its reliability by calculating Dice coefficient (Dice) and Average Hausdorff Distance (AHD) for predefined areas in 100 CT scans from ischaemic stroke patients.

**Results:**

The resulting CT/MRI templates were age and sex matched to a general stroke population (median age 71.9 years (62.1–80.2), 60% male). Specifically, this accounts for relevant structural changes related to aging, which may affect registration. Applying the validated MRI to CT alignment (Dice > 0.78, Average Hausdorff Distance < 0.59 mm) resulted in our final CT-MRI atlas. The atlas has 52 manually segmented regions and covers the whole brain. The alignment of four cortical and subcortical brain regions with our CT-normalisation pipeline was reliable for small/medium/large infarct lesions (Dice coefficient > 0.5).

**Conclusion:**

The newly created CT-MRI brain atlas has the potential to standardise stroke lesion segmentation. Together with the automated normalisation pipeline, it allows analysis of existing and new datasets to improve prediction tools for stroke patients (free download at https://forms.office.com/r/v4t3sWfbKs).

**Supplementary Information:**

The online version contains supplementary material available at 10.1007/s00234-021-02875-9.

## Introduction

In order to translate changes from neuroimaging studies to a general population, it is important to perform analysis on a group level, which requires spatial normalisation of individual data [1, 2]. Most contemporary spatial normalisation algorithms are guided by template images from a neurologically healthy and, notably, young population. Due to morphological and volumetric changes that occur with aging [3], studies focusing on disorders that affect elderly populations should use age-specific templates as this improves segmentation results [4, 5].

Computed tomography (CT) is the most commonly used imaging modality in acute stroke with essentially all patients undergoing a CT scan as part of the acute assessment. Although CT involves exposure to ionising radiation, has limited sensitivity in the ultra-acute stage of ischaemic stroke and is less sensitive in some specific stroke subtypes such as brainstem infarction, there are several key advantages of CT over MRI. These advantages include speed, cost, availability and safety, with no absolute contraindications to CT, which makes it the primary modality for treatment decisions and early prognostic assessments in acute stroke [6].

In contrast to the clinical setting, most stroke outcome prediction studies include MRI scans only, which limits the generalisability. In recent years, a small number of CT-based predictive studies regain attention (e.g. [7, 8]).

Ischaemic lesion volume and location have been described as key prognostic factors in stroke recovery [9]. Despite this, there is to date no universally agreed and well-validated standard anatomical atlas which can be used reliably for stroke lesion segmentation in clinical practice or in research studies.

While the existing and most-often used atlases describe brain anatomy in detail, they may not capture stroke population–specific anatomy [10], are time consuming to generate and are susceptible to inter- and intra-rater variability [11] especially when used—as commonly done—without any further validation.

This is also true for the validation of the alignment of individual CT images to template space. Whereas research tools to normalise MRI scans are well established and validated, tools for accurate normalisation and co-registration of CT images are limited. As such, CT-based predictive studies use non-standard methods to register CT images to a template/atlas, generally without reporting formal validation of the chosen method. This is of concern, not only because image normalisation has been mostly performed based on templates developed from young and healthy adults, but also because commonly used algorithms often fail to successfully normalise CT scans [12].

Given the variety of ischaemic stroke patterns, large datasets are necessary to develop robust and comprehensive predictive models. To achieve this, an important prerequisite is a standardised location identifier, which allows researchers to analyse large datasets in an efficient, inexpensive and standardised way.

We therefore aimed to create a reasonably detailed anatomical whole-brain CT and MRI atlas, which is based on stroke population–specific templates, comes with a semi-automated validated registration algorithm for CT and is freely available. This tool could then be used in both well resourced and more challenging research environments. Such a research tool has the potential to facilitate and standardise clinical research, including mixed modality studies, and thereby improve stroke outcome prediction.

## Methods

The collection of CT scans was approved by the Royal Melbourne Hospital Human Research Ethics Committee with no individual patient consent required. The AIBL study was approved by the institutional ethics committees of Austin Health, St Vincent’s Health, Hollywood Private Hospital and Edith Cowan University, and all volunteers gave written informed consent before participating [13].

Templates, atlas and registration algorithm are available from https://forms.office.com/r/v4t3sWfbKs. Further data is available from the corresponding author upon reasonable request, including a data-sharing agreement.

**Fig. 1:**
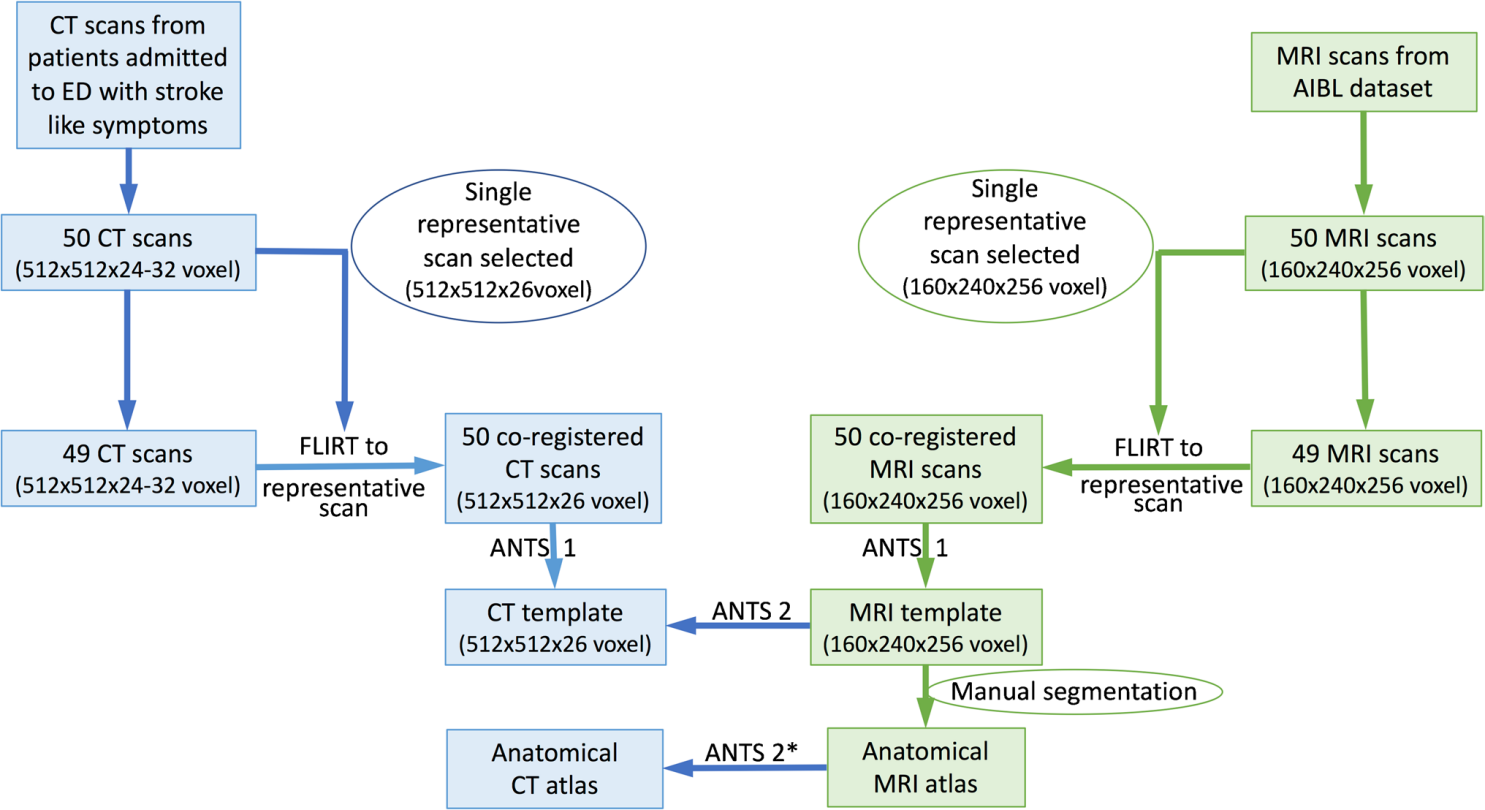
Workflow templates and atlas; FLIRT (FMRIB’s Linear Image Registration Tool): linear affine registration with 12 degrees of freedom; ANTS 1: affine and non-linear registration with 60 × 90 × 40 iterations and cross correlation as similarity matrix; ANTS 2: affine and non-linear registration with 60 × 90 × 40 iterations and point-set expectation and mutual information as similarity matrix; * plus manual adaptation

The overall workflow for creation of the CT template and atlas (in blue) and the MRI template and atlas (in green) is shown in Fig. 1: Through reviewing of CT scans of patients with stroke-like symptoms and filtering of the AIBL database for MRI scans, respectively, 50 normal-for-age CT and MRI scans were collected, and one representative CT and MRI scan was selected. The remaining 49 CT scans and 49 MRI scans were then registered to the respective representative scan using FMRIB’s Linear Image Registration Tool (FLIRT).

Furthermore, affine and non-linear registration with 60 × 90 × 40 iterations and cross correlation as similarity matrix was performed for the 50 co-registered CT scans and the 50 co-registered MRI scans independently, which resulted in the final CT and MRI template. Finally, the MRI template was manually segmented to produce a whole-brain neuroanatomical MRI atlas. As a last step, this atlas was transformed into CT template space using affine and non-linear registration with 60 × 90 × 40 iterations and point-set expectation and mutual information as similarity matrix (further details below).

### Data collection

We reviewed in random sequence CT scans acquired between 2008 and 2014 in suspected stroke patients aged 55 to 95 where the final diagnosis was not stroke and where the CT scan was normal for age according to the radiology report as part of standard clinical care, until we identified 50 such scans. We collected images by age groups (8 images from patients aged 50–59 years, 13 images from patients aged 60–69 years, 16 images from patients aged 70–79 years, 12 images from patients aged 80–89 years, 1 image from a patient aged 95 years) to derive an age- and sex-matched cohort to participants from A Very Early Rehabilitation trial (AVERT, *n* = 2104, [14]) which was taken as representative of a general stroke population [15]. All 50 CT scans (SIEMENS Sensation 16 CT scanner, in-plane resolution = 0.38–0.45 mm, helical/sequential with 280/360 mAs, 120 kV, 1 s rotation time, 4.5–5 mm slice thickness) were subsequently reviewed by two neurologists (TK, 5 years of experience; NY, 6 years of experience) to confirm the absence of hyper/hypodensities and any other structural abnormalities. Well-known age-related changes, e.g. widening of the ventricles and white matter changes, were permitted.

We filtered the AIBL dataset (The Australian Imaging, Biomarkers & Lifestyle Flagship Study of Ageing; www.aibl.csiro.au; [13]) for cognitively normal participants, i.e. people without any cognitive symptoms suspicious for mild cognitive impairment or any form of dementia during a follow-up period of 54 months (part of the AIBL study), by age group to collect T1-weighted images (SIEMENS TrioTim: 3 Tesla, SIEMENS Verio: 3 Tesla, SIEMENS Avanto: 1.5 Tesla, sequence: MPRAGE, FOV 208 × 240x256mm, reconstructed resolution: 1 × 1 × 1 mm, TE = 2.98 ms, TR = 2300 ms, TI = 900 ms) from 50 people age and sex matched to the AVERT stroke population.

### Building of template

After visual selection of a representative CT image, we performed affine registration of all other CT scans to the representative image FLIRT (version 6·0, Oxford, UK). FLIRT is a fully automated robust and accurate tool for intra- and inter-modal brain image registration [16–18]. With the 50 resulting co-registered scans, we created a template using Advanced Normalisation Tools (ANTs) with 60 × 90 × 40 iterations per registration and cross correlation as a similarity metric. An identical approach was used to build the MRI template using T1 images, to assist with neuroanatomical atlas development in this template space.

Finally, as the CT and MRI images did not originate from the same individuals, we compared the ventricular size of the 50 CT images versus the 50 MRI images, which were used to build the templates by using *t*-test.

### CT normalisation algorithm and validation

In order to maintain consistency and given it was the most reliable tool for MRI [19], we used the Advanced Normalisation Tools (ANTs) to develop a normalisation pipeline, which allows normalisation of standard-resolution CT scans to our standard-resolution CT template. This was a methodologically challenging exercise. We investigated the effect of adjusting the variables mentioned below (which have been shown to improve MRI normalisation) in both individual images and in the template:

- With/without skull
- With/without gantry correction
- Set origin to anterior commissure
- With/without whole-brain/template mask
- With/without lesion mask.

#### Validation

First, we visually reviewed the individual registered images for proper orientation. As a formal validation, we defined four fiducial areas per hemisphere, which can be reliably defined in most clinical CT scans even if the image quality is low: the insular ribbon (cortical, supratentorial), the cerebellum (infratentorial), the caudate nucleus (subcortical) and the anterior horn of the lateral ventricle.

These regions were manually segmented by a neurologist (TK) in a randomly selected group of 100 CT scans, stratified by infarct volume size (small (< 10 ml), medium (10–70 ml) and large (> 70 ml) ischaemic lesions). One hundred stroke images were selected from seven AVERT sites in Australia and one site in New Zealand. For this, we reviewed available AVERT imaging at random until we reached 100 representative infarct lesions, including small (*n* = 33), medium (*n* = 34) and large infarcts (*n* = 33).

In 10% of images, a second neurologist (VT, > 15 years of experience) performed the same segmentation and inter-rater reliability was tested comparing the overlap of two regions of interest with the Dice coefficient (Dice) and measuring the distance of the boundaries of two regions of interest (Average Hausdorff Distance (AHD)[20]).

The alignment between the manually segmented individual’s brain structures and the same structures in the CT template was also evaluated using the Dice and the AHD.

As the registration of individual MRI scans and templates is well established and validated [19] with the software used in this study (ANTs), we did not perform a formal validation of individual MRI to MRI template registration.

### Neuroanatomical brain segmentation (i.e. parcellation of the brain)

**Fig. 2:**
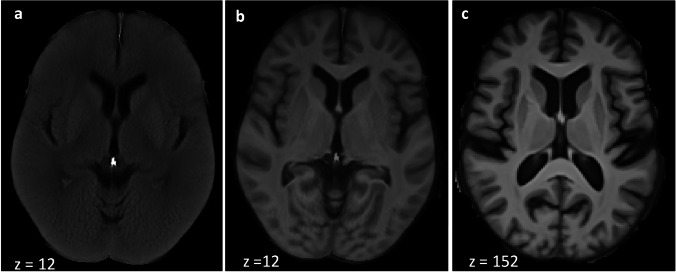
**a** CT template (512 × 512 × 26 voxel), **b** MRI-CT template (512 × 512 × 26 voxel), **c** MRI template (160 × 240 × 256 voxel).

As the resolution of the CT template was insufficient for anatomically accurate segmentation (see Fig. 2a), we manually segmented the whole brain based on the MRI template using the software-tool ITK-SNAP [21]. Based on the cortical region definitions from the adapted Desikan-Killiany-Tourville labelling protocol [19], we segmented the brain into 52 regions (see Table 1). In contrast to the definition used in the Desikan-Killiany-Tourville labelling protocoll, we included to the cortex adjacent white matter (WM) and therefore labelled the different areas as ‘regions’ and not ‘cortex’. Anatomical landmarks were verified using the Cortical Delineation Protocol [22].

**Table 1:** The atlas comprises 52 brain regions. Eloquent areas as described in previous stroke outcome studies are marked in bold

• Frontal lobe (8 regions)
**- Superior frontal region (SFR), middle frontal region (MFR), inferior frontal region (IFR)** subdivided into pars opercularis (IFR-op), pars triangularis (IFR-t) and pars orbitalis (IFR-or), orbitofrontal region (OFR) with a lateral division (OFR-l) and a medial division (OFR-m), **precentral region (PR)**, paracentral region (PaR), other frontal WM
- Accumbens area
**• Parietal lobe** (6 regions)
**- Postcentral region (PoR)**, supramarginal region (SMR), superior parietal region (SPR), inferior parietal region (IPR), **precuneal region,** other parietal WM
• Temporal lobe (9 regions)
**- Superior temporal region (STR),** middle temporal region (MTR), **inferior temporal region (ITR)**, transverse temporal region (TTR), entorhinal region, fusiform region, other temporal WM
- Hippocampus, parahippocampus
• Occipital lobe (5 regions)
- Lingual region (LR), pericalcarine region, cuneal region (CR), lateral occipital region (LOR), other occipital WM
• Cingulate region (3 regions)
- Anterior cingulate region (ACR, rostral and caudal), posterior cingulate region (PCR), isthmus cingulate
• Corpus callosum (CC)
**• Insular region (IR)**
• Subcortical structures (7 regions)
- Caudate nucleus, **putamen,** lentiform nucleus, pallidum**, thalamus,** amygdala, other WM
• CST (7 regions)
- Corona radiata (CR anterior, posterior and superior), **internal capsule** (anterior (ALIC), posterior limb (PLIC) and retrolenticular part (RLIC)), external capsule (EC), superior longitudinal fasciculus (SLF), superior fronto-occipital fasciculus, uncinate fasciculus (UF), other corticospinal tracts
• Cerebellum, vermis cerebelli
**• Brainstem** (3 regions)
- Mesencephalon, pons, medulla oblongata

Brain structures prognostically important for stroke outcome and which could not be defined on the newly created MRI template (T1), particularly corticospinal tracts (CST), were incorporated by registering selected predefined white matter tracts from the Johns Hopkins University (JHU) ICBM-DTI-81 white matter labels atlas [23] to the MRI template (see below).

The manual segmentation was performed by a neurologist (TK). In the case of uncertainty, consensus was achieved between an expert panel including a highly experienced neuroradiologist (PMD, > 30 years of experience) following discussion.

Using ANTs, we aligned the MRI template onto the CT template to obtain the identical atlas for the standard-resolution CT. This alignment was tested by defining 5 fiducial areas (caudate nucleus, lentiform nucleus, thalamus, lateral ventricles, 2 upper brain slices (all bilateral)) in the CT and MRI template (manual segmentation by a neurologist (TK) in both templates) and quantifying their overlap calculating Dice and AHD.

## Results

### CT and MRI templates

The collected 50 standard-resolution (512 × 512 × 24–32) healthy CT brain scans were obtained from patients (median age 71.9 years (62.1–80.2), 60% male), who represent a general stroke population. According to their discharge letters, the stroke-like symptoms were due to transient ischaemic attacks, seizures (without known epileptic disorder), migraine, benign paroxysmal vertigo or functional disorders. The CT template has a resolution of 512 × 512 × 26 voxels with a voxel size of 0.45 × 0.45 × 5 mm.

The collected 50 high-resolution (160 × 240 × 256) healthy MRI brain scans were obtained from participants (median age 71.9 years (62.1–80.2), 60% male), which are sex and age matched to the stroke population. The MRI template has a resolution of 160 × 240 × 256 voxels with a voxel size 1.2 × 1 × 1 mm.

There was no significant difference in the ventricle sizes of the CT versus the MRI scans (CT: 35.7 ml, MRI: 33.7 ml, *p* > 0.05, *t*-test), which shows comparability of the two templates in terms of brain health relative to age.

### CT normalisation algorithm and validation

Affine and subsequently affine and non-linear registration with cross correlation as a similarity metric produced the most robust CT to CT template normalisation results (see repository https://forms.office.com/r/v4t3sWfbKs for full algorithm).

Normalisation of skull-stripped images [24] led to severe visual artifacts. Neither gantry tilt correction nor setting the origin of the individual images and the template to the anterior commissure (as close as feasible in 5 mm CT) improved the resultant registrations. Those steps were therefore not included in the final algorithm. As expected, the use of a manually drawn infarct lesion mask did improve the result especially in large lesions, whereas the addition of a whole-brain mask had no added value.

**Fig. 3:**
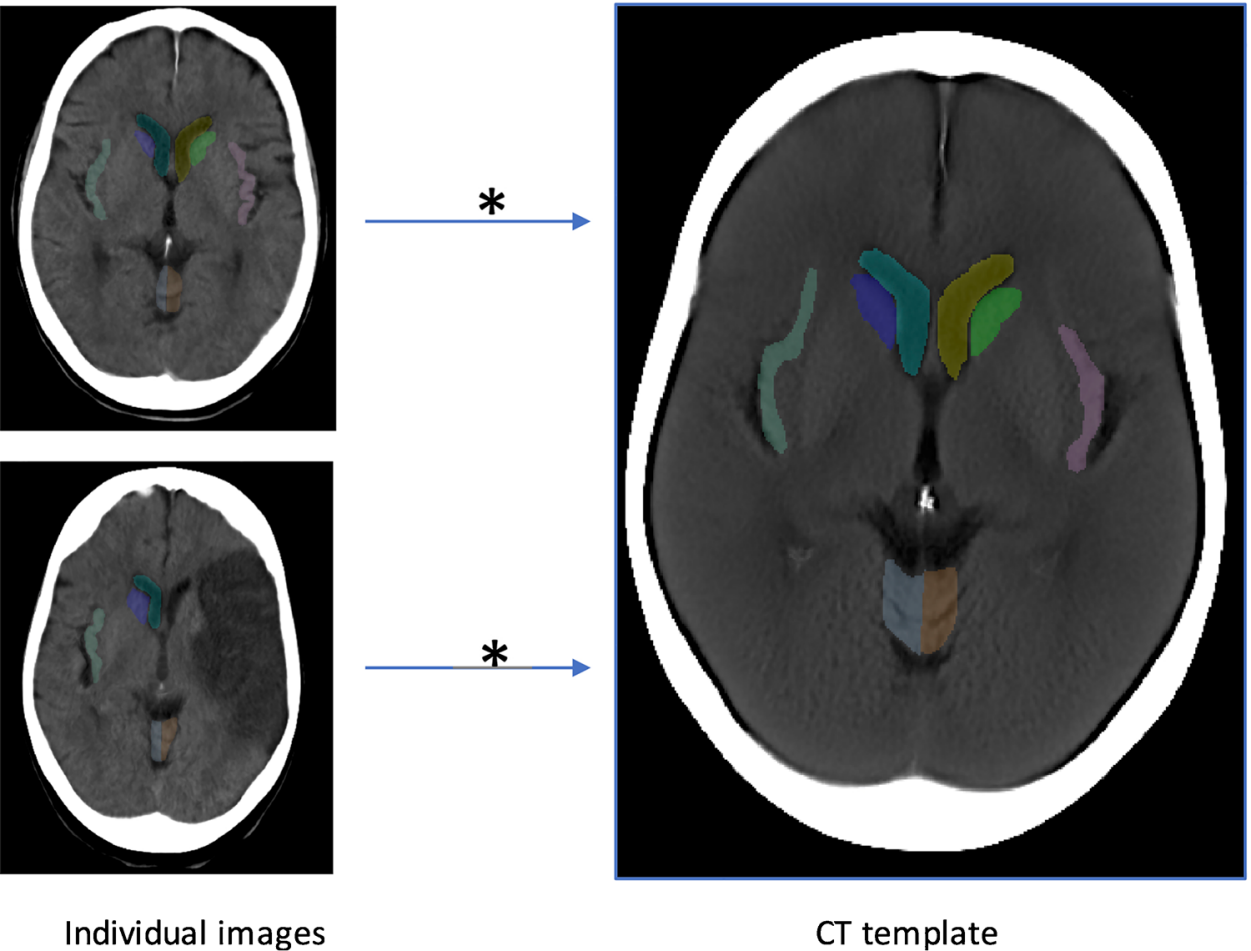
Validation of alignment with the newly developed registration pipeline (*)

The alignment between the individual images and the template was reliable as shown by Dice > 0.5 and AHD (see Table 2 and Fig. 3). There was no major difference in the performance of the algorithm for images with small (< 10 ml), medium (10–70 ml) or large lesions (> 70 ml; maximum 349 ml; for further details in the different types of stroke included, see online supplement). There did not appear to be a critical upper ischaemic lesion volume which resulted in severe misregistration.

**Table 2:** Dice scores (Dice) and Average Hausdorff Distance (AHD) of 8 regions between the individual images and the CT template

**Region of interest**	**Dice** **Median (IQR)**	**AHD **[25] **Median (IQR)**
Caudate L	0.72 (0.09)	0.40 (0.31)
Caudate R	0.70 (0.12)	0.47 (0.36)
Lateral ventricle, ant horn L	0.79 (0.11)	0.36 (0.88)
Lateral ventricle, ant horn R	0.84 (0.10)	0.30 (0.31)
Cerebellum L	0.78 (0.05)	0.36 (0.12)
Cerebellum R	0.77 (0.07)	0.36 (0.16)
Insular ribbon L	0.61 (0.11)	1.01 (0.68)
Insular ribbon R	0.62 (0.09)	0.78 (0.46)

### CT and MRI atlas

The newly created MRI atlas covers the whole brain and defines the 52 regions as described above (see Table 1).

The described templates and atlas in combination with the normalisation algorithm (script) are freely available (with citation of the current manuscript) and can be downloaded using the following link: https://forms.office.com/r/v4t3sWfbKs (for research purpose only).

### MRI to CT registration and validation

**Fig. 4:**
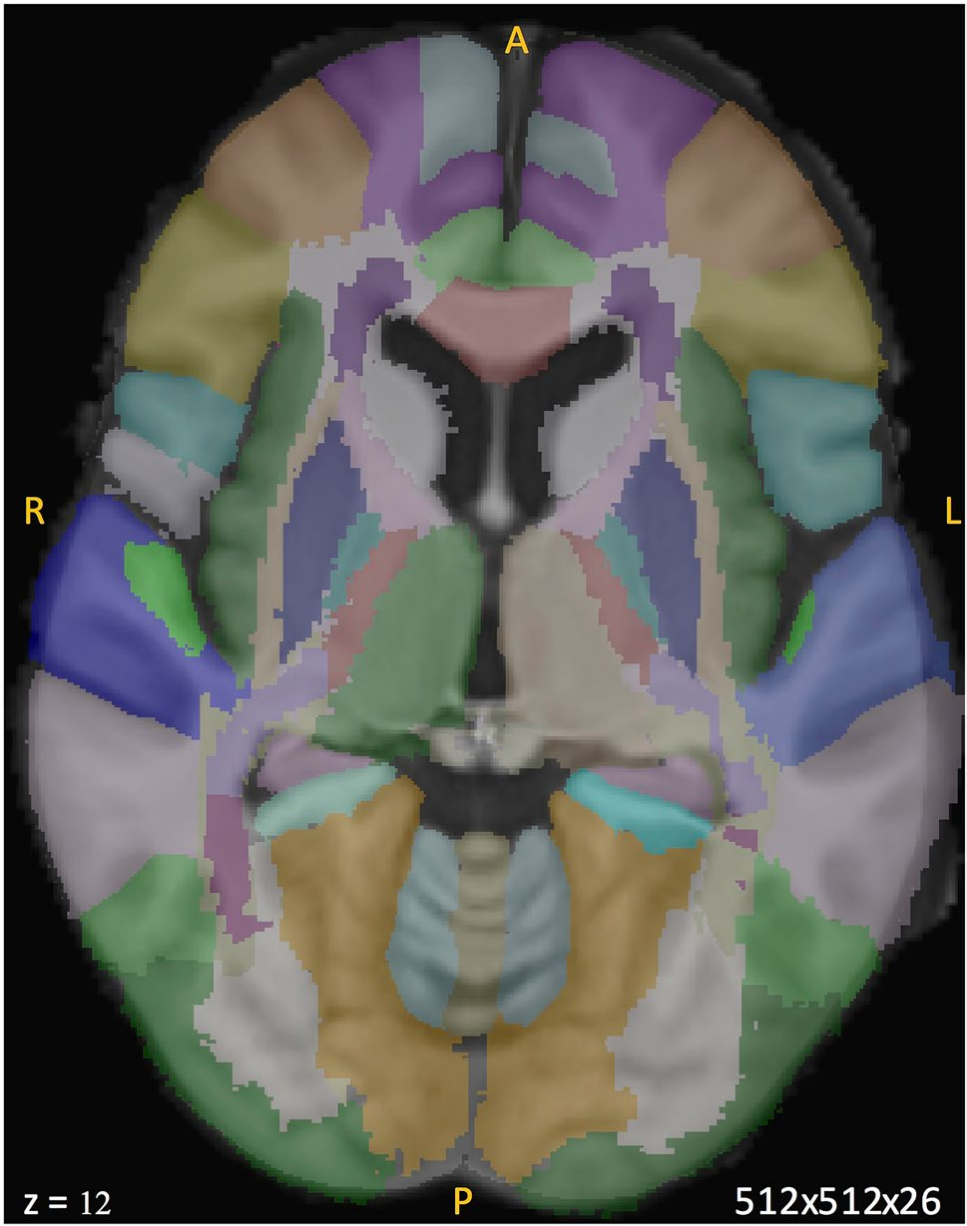
Example axial view (slice 1) of the cortical and subcortical regions of the CT-MRI atlas

To transfer this atlas onto CT, we aligned the high-resolution MRI template to the standard-resolution CT template (Fig. 4). Whereas the registration of CT images to MRI images (in general of the same individual) is well established, the inverse process was more challenging. Ultimately, the manual segmentation of eight areas in the CT and MRI templates together with a combination of point-set expectation (80%) and mutual information (20%) as similarity metrics with 60 × 90 × 40 iterations were used for the MRI to CT registration with a resulting low-resolution MRI template (512 × 512 × 26 voxels). Neither suggested MRI landmarks (fiducial points) for validation [26] nor their proposed adaptation for CT scans [12] were useful as they could not be clearly identified in both templates due to the differing resolution. Therefore, we validated the registration comparing the alignment of four fiducial areas, which could be defined confidently in both the standard 5 mm CT slices and 1 mm MRI slices. Dice and AHD showed excellent alignment on a level < 1 mm (see Table 3):

**Table 3:** Dice score (Dice) and Average Hausdorff Distance (AHD) for different brain regions to validate alignment of MRI to CT template

Brain region	Dice Median (IQR)	AHD [mm] Median (IQR)
All	0.88	
Caudate nucleus right	0.82	0.250
Caudate nucleus left	0.85	0.170
Lentiform nucleus left	0.82	0.261
Lentiform nucleus right	0.82	0.243
Lateral ventricles	0.79	0.587
Thalamus right	0.79	0.303
Thalamus left	0.79	0.292
Upper brain slices (above lateral ventricles)	0.89	0.272

## Discussion

We have developed a neuroanatomical CT-MRI atlas, which is based on a stroke population–specific template (age and sex matched to a standard stroke population) with an automated, reliable and validated CT normalisation algorithm. Importantly, the atlas covers the whole brain and comprises regions which are clinically and prognostically relevant.

The combination of an identical CT and MRI atlas allows this method to be used to analyse multimodal datasets and thereby to study large, clinically relevant samples. This freely available tool has the potential to standardise and facilitate lesion location–based research in stroke in a variety of research environments and thereby to improve stroke outcome prediction significantly.

The main hurdle to performing predictive research on clinically acquired scans (mainly CT scans) is the absence of a standardised research tool. While two well-designed CT templates for an elderly population with automated co-registration pipelines exist [12, 27], none of them includes a matching atlas.

Currently, many predictive studies use imprecise visual rating scores, with the Alberta Stroke Programme Early CT Score (ASPECTS, [28]) being the most frequently used (e.g. [29, 30]), or a variety of different atlases, which are based on MRI templates/brains of young healthy adults.

For example, Ernst and colleagues [7] used the Laboratory of Neuro Imaging Probabilistic Brain Atlas (LPBA) [31] and manually added 5 cerebral structures to cover the whole brain, resulting in 66 brain areas in total for their predictive CT-based study. Cheng et al. (2014) [32], Yassi and colleagues (2015, total of 132 structures) [33] and Wu et al. (2015) [34] combined the Harvard–Oxford Cortical Structural Atlas [35] and the John Hopkins University International Consortium of Brain Mapping Diffusion Tensor Imaging (JHU ICBM-DTI)-81 White Matter Labels atlas [36]. Hope et al. (2013) [37] extracted a total of 232 cortical and subcortical regions of interest based on the Anatomy Toolbox [38] and the JHU ICBM-DTI-81, whereas Munsch and colleagues (2016) [39] identified eloquent regions from the Automated Anatomic Labeling (based on the MNI single-subject MRI brain) [40] Brodmann, and JHU ICBM-DTI 81 atlases (all MRI studies). Adapting those atlases to cover the whole brain and to fit the studied population is very time consuming. Furthermore, the large variability in input data (between 66 and 232 regions of interest) and the omission of potentially eloquent areas known to be critical in stroke recovery limit the comparability of results. In addition, none of those studies addressed the handling of overlap between different brain regions, which occurs when combining multiple atlases, nor the overlay of an atlas based on young healthy brains onto aging brains nor the use of MRI based atlases on CT without validated alignment.

The atlas presented here covers the whole brain and thereby prevents the necessity of combining different atlases with subsequent overlap of certain areas or manual adaptation of atlases. Furthermore, in contrast to the commonly used atlases, our population-specific atlas accounts for age-specific changes, including enlarged ventricles, widened sulci and cortical atrophy, and the subsequent volumetric changes of eloquent areas of interest, and allows direct comparison of results from different studies.

Rorden and colleagues presented a stroke population–specific CT and MRI template and the corresponding validated registration algorithm [12]. While these templates have been well developed and a thorough validation of the proposed normalisation has been performed, they are implemented into a Statistical Parametric Mapping (SPM) toolbox and therefore are only usable with a Matlab license. More recently, a freely available high-resolution CT and T2-weighted Fluid Attenuated Inversion Recovery (FLAIR)-MRI template based on an elderly population has been published with a corresponding processing pipeline [27]. It also represents the brain in great detail and has been brought into Montreal Neurological Institute (MNI) space with limited validation on clinical data. While this makes it possible to overlay MNI-space-atlases, the utility of these atlases in stroke studies is limited as most do not cover the whole brain or are too granular in order to develop clinically meaningful predictive models.

These two existing stroke population–based CT templates have high resolution. Normalisation to those templates can be very time consuming especially when computational power is limited and may require excessive data interpolation for standard-resolution CT scans, especially when thin slice (1 mm) CT scans have not been acquired. Therefore, we have chosen a standard-resolution CT template. This results in the challenge that commonly used landmarks are often not captured due to the 5 mm slice thickness. Furthermore, we have taken individual CT scans from a purely clinical dataset of acute stroke patients (AVERT, [14], recruitment 2008–2014) to validate the normalisation algorithm. These scans have been acquired at different sites with different scanner protocols and are often of ‘real world’ quality including some movement artifacts. This made the development of an automated normalisation pipeline particularly challenging and required two different registration steps. Hence, our normalisation algorithm is very robust and is capable of coregistering images of variable qualities reliably regardless of aforementioned variations. It is therefore especially helpful for large datasets with multiple image acquisition sites.

Given the massive diversity of stroke lesion patterns, patient characteristics and therapy approaches, one major limitation of current studies focusing on general outcome prediction after stroke using neuroimaging markers is the limited number of included patients. Allowing for the analysis of CT imaging allows the inclusion of additional and existing large datasets (e.g. Virtual Trials Archives (VISTA, http://www.virtualtrialsarchives.org)) for research and would ensure generalisability, especially as CT scans are, and most likely will remain, the imaging modality of choice in acute stroke patients. This is particularly relevant with the increasing number of hyperacute stroke trials in recent years [41–45], as well as with a view towards allowing groups from medium- and low-income settings to participate in important stroke outcome prediction research.

Despite decades of research, a generalisable, clinically adopted stroke outcome prediction model is not available yet. Our newly created CT-MRI atlas has the potential to facilitate research in stroke outcome prediction with a particular focus on CT-based research. It pioneers the way for efficient analysis of large multimodal data sets and, once adapted into research practice, provides an opportunity to pool and directly compare data from different studies. It covers the entire brain, avoiding ‘undefined’ areas, and focuses on a limited number of clinically relevant structures. We believe that our atlas is granular enough for stroke outcome prediction research, while being simple enough to provide results which are clinically meaningful.

Future studies employing our methodology and using multimodal CT (including CTP) could further explore the association between penumbral location, reperfusion success and clinical outcome after acute reperfusion therapy.

This study has some limitations. The CT template appears visually blurred and has limited grey-white matter differentiation, which results in only few anatomical landmarks being clearly identifiable. Given that we visually assessed each registration step carefully and at every iteration the individual images appeared acceptable, we suspect the blurring is related to the inter-individual variance in brain anatomy and the clinical nature of the scans, rather than poor registration. This blurring did result in a methodological challenge as we were unable to directly ascertain the accuracy of the atlas on the CT template. However, given that the accuracy of the registration of the MRI to CT template was excellent for the tested subcortical and cortical structures and given that the alignment of individual CT scans to the CT template was reliable, we believe that the resultant CT atlas has adequate precision for its stated purpose.

By design, our CT template is not perfectly symmetrical. This should be taken into account when implementing the template, but does not limit its use as standard location identifier.

Unlike the other atlas structures, the CSTs were imported from an existing standard atlas based on young and healthy brains, as this was the only method to reliably identify this structure. We chose this approach as we believe the known prognostic importance of the CSTs for stroke outcome prediction outweighs this potential imprecision. Nevertheless, we welcome efforts to develop a white matter tract atlas based on diffusion tensor imaging from the aging (stroke population specific) population, which could be adapted into the current CT-MRI atlas.

Determining the number of individual images to build a template is a challenge in this area of research, which has been only recently begun to be addressed in studies (e.g. [46]). In light of this research gap, we have determined the numbers of scan used to build the templates based on comparable templates (e.g. [12, 27, 47, 48]).

## Conclusion

The newly created stroke population–specific whole-brain CT-MRI atlas together with the automated, reliable and validated CT normalisation algorithm makes large multimodal clinical datasets easily accessible for research. As a freely available atlas (https://forms.office.com/r/v4t3sWfbKs), which covers the whole brain without being granular, it has the potential to standardise and facilitate lesion location–based research in stroke in variably resourced research environments which may facilitate its adoption into research practice.

## Supplementary Information

Below is the link to the electronic supplementary material.

Supplementary file1 (DOCX 30 KB)
