# Supplementary material for: Stroke population–specific neuroanatomical CT-MRI brain atlas
Source: Neuroradiology. 2022 Jan 30;64(8):1557–67. doi: 10.1007/s00234-021-02875-9 (PMC9271109; doi:10.1007/s00234-021-02875-9)
Supplement: Supplementary file 1 — Supplementary file1 (DOCX 30 KB) [file 234_2021_2875_MOESM1_ESM.docx]

| **ID for validation** | **Time between symptom onset and imaging [hours]** | **OXFORD-SHIRE CLASSI-FICATION** | **Infarct location** | **Total ischemic lesion volume [mm^3^]** |
| --- | --- | --- | --- | --- |
| 1 | 3.6 | PACI | Left hemisphere | 5.9 |
| 2 | 2.12 | PACI | Both hemispheres | 92.4 |
| 3 | 1.73 | PACI | Right hemisphere | 110.4 |
| 4 | 2.57 | PACI | Both hemispheres | 134.4 |
| 5 | 14.8 | PACI | Left hemisphere | 27.6 |
| 6 | 3.53 | LACI | Right hemisphere | 8.2 |
| 7 | 3.1 | TACI | Left hemisphere | 105.6 |
| 8 | 3.23 | TACI | Right hemisphere | 113.8 |
| 9 | 2.0 | PACI | Left hemisphere | 8.3 |
| 10 | 15.13 | PACI | Right hemisphere | 136.4 |
| 11 | 2.72 | PACI | Brainstem, right cerebellum, both hemispheres | 58.8 |
| 12 | 5.62 | PACI | Right hemisphere | 85.8 |
| 13 | 2.47 | TACI | Left hemisphere | 19.2 |
| 14 | 5.05 | PACI | Right hemisphere | 114.2 |
| 15 | 5.73 | POCI | Right cerebellum, right hemisphere | 25.0 |
| 16 | 2.67 | PACI | Right hemisphere | 77.6 |
| 17 | 3.95 | POCI | Right cerebellum, right hemisphere | 28.4 |
| 18 | 2.53 | PACI | Left hemisphere | 75.9 |
| 19 | 1.92 | TACI | Right hemisphere | 41.9 |
| 20 | 2.9 | TACI | Both hemispheres | 191.9 |
| 21 | 10.5 | PACI | Left hemisphere | 6.8 |
| 22 | 3.8 | POCI | Left hemisphere | 9.2 |
| 23 | 1.47 | PACI | Right hemisphere | 1.8 |
| 24 | 1.92 | TACI | Right hemisphere | 60.0 |
| 25 | 1.9 | TACI | Right hemisphere | 119.6 |
| 26 | 9.25 | PACI | Brainstem, both hemispheres | 181.8 |
| 27 | 2.58 | POCI | Left hemisphere | 98.4 |
| 28 | 12.47 | PACI | Both hemispheres | 163.3 |
| 29 | 2.72 | TACI | Left cerebellum, left hemisphere | 99.1 |
| 30 | 7.63 | TACI | Brainstem, left hemisphere | 230.1 |
| 31 | 5.22 | TACI | Brainstem, right hemisphere | 281.8 |
| 32 | 3.2 | PACI | Brainstem, both hemispheres | 287.4 |
| 33 | 1.87 | TACI | Right hemisphere | 77.1 |
| 34 | 1.83 | PACI | Left hemisphere | 29.1 |
| 35 | 2.25 | TACI | Brainstem, right hemisphere | 170.7 |
| 36 | 2.22 | PACI | Right hemisphere | 73.4 |
| 37 | 7.81 | TACI | Brainstem, right hemisphere | 193.0 |
| 38 | 1.83 | PACI | Left hemisphere | 90.1 |
| 39 | 2.1 | TACI | Left hemisphere | 2.9 |
| 40 | 19.0 | POCI | Both hemispheres | 91.9 |
| 41 | 1.78 | TACI | Left hemisphere | 26.5 |
| 42 | 2.18 | LACI | Both hemispheres | 5.3 |
| 43 | 10.95 | PACI | Both hemispheres | 0.8 |
| 44 | 3.67 | POCI | Left hemisphere | 15.6 |
| 45 | 1.05 | PACI | Left hemisphere | 1.2 |
| 46 | 2.37 | TACI | Cerebellum, both hemispheres | 64.2 |
| 47 | 7.13 | LACI | Both hemispheres | 1.1 |
| 48 | 2.15 | TACI | Left hemisphere | 95.3 |
| 49 | 3.62 | LACI | Right hemisphere | 6.0 |
| 50 | 2.17 | POCI | Right hemisphere | 1.6 |
| 51 | 2.42 | TACI | Right hemisphere | 3.4 |
| 52 | 2.13 | TACI | Both hemispheres | 1.9 |
| 53 | 2.07 | PACI | Right hemisphere | 1.8 |
| 54 | 19.28 | PACI | Right hemisphere | 120.5 |
| 55 | 5.87 | POCI | Right hemisphere | 4.6 |
| 56 | 1.82 | TACI | Right hemisphere | 0.2 |
| 57 | 9.43 | PACI | Right hemisphere | 7.4 |
| 58 | 3.6 | PACI | Both hemispheres | 7.8 |
| 59 | 1.37 | TACI | Right hemisphere | 3.3 |
| 60 | 1.7 | PACI | Right hemisphere | 0.4 |
| 61 | 16.62 | PACI | Right hemisphere | 18.4 |
| 62 | 1.73 | TACI | Both hemispheres | 99.5 |
| 63 | 2.87 | PACI | Brainstem, right hemisphere | 52.0 |
| 64 | 1.58 | LACI | Right hemisphere | 2.2 |
| 65 | 18.5 | TACI | Both hemispheres | 226.4 |
| 66 | 3.68 | PACI | Both hemispheres | 57.1 |
| 67 | 2.73 | POCI | Right hemisphere | 0.8 |
| 68 | 3.75 | LACI | Left hemisphere | 184.6 |
| 69 | 2.12 | PACI | Brainstem, right hemisphere | 33.1 |
| 70 | 2.72 | TACI | Right hemisphere | 33.7 |
| 71 | 2.08 | PACI | Right hemisphere | 53.9 |
| 72 | 2.33 | PACI | Left hemisphere | 14.1 |
| 73 | 8.2 | PACI | Right hemisphere | 13.3 |
| 74 | 3.27 | PACI | Left hemisphere | 10.1 |
| 75 | 21.28 | TACI | Right hemisphere | 88.0 |
| 76 | 3.8 | PACI | Right hemisphere | 6.2 |
| 77 | 2.82 | PACI | Both hemispheres | 41.7 |
| 78 | 5.63 | PACI | Left hemisphere | 5.7 |
| 79 | 2.53 | PACI | Both hemispheres | 15.8 |
| 80 | 2.97 | POCI | Left hemisphere | 4.4 |
| 81 | 6.3 | LACI | Left hemisphere | 4.7 |
| 82 | 2.1 | PACI | Right hemisphere | 16.9 |
| 83 | 10.88 | PACI | Right hemisphere | 2.4 |
| 84 | 3.78 | PACI | Right hemisphere | 46.7 |
| 85 | 1.78 | PACI | Left hemisphere | 5.9 |
| 86 | 6.72 | POCI | Left hemisphere | 0.6 |
| 87 | 1.95 | POCI | Brainstem, both hemispheres | 131.3 |
| 88 | 5.48 | LACI | Brainstem, right cerebellum, both hemispheres | 3.9 |
| 89 | 2.3 | LACI | Left hemisphere | 3.4 |
| 90 | 2.37 | TACI | Brainstem, right cerebellum, right hemisphere | 196.3 |
| 91 | 2.05 | LACI | Right hemisphere | 43.2 |
| 92 | 1.88 | TACI | Right hemisphere | 24.9 |
| 93 | 2.58 | TACI | Brainstem, left cerebellum, left hemisphere | 13.7 |
| 94 | 2.45 | PACI | Right hemisphere | 14.4 |
| 95 | 1.53 | PACI | Right hemisphere | 12.0 |
| 96 | 1.8 | LACI | Right hemisphere | 17.6 |
| 97 | 2.1 | PACI | Right hemisphere | 13.8 |
| 98 | 8.27 | PACI | Right hemisphere | 39.2 |
| 99 | 4.63 | PACI | Brainstem, both hemispheres | 61.5 |
| 100 | 2 | PACI | Both hemispheres | 125.3 |

Supplement Table A: Type of ischaemic strokes included for validation of CT-CT-normalization pipeline; LACI = lacunar circulation infarct, PACI = partial anterior circulation infarct, POCI = posterior circulation infarct, TACI = total anterior circulation infarct
